# Susceptibility to corticosteroid-induced adrenal suppression: a genome-wide association study

**DOI:** 10.1016/S2213-2600(18)30058-4

**Published:** 2018-06

**Authors:** Daniel B Hawcutt, Ben Francis, Daniel F Carr, Andrea L Jorgensen, Peng Yin, Naomi Wallin, Natalie O'Hara, Eunice J Zhang, Katarzyna M Bloch, Amitava Ganguli, Ben Thompson, Laurence McEvoy, Matthew Peak, Andrew A Crawford, Brian R Walker, Joanne C Blair, Jonathan Couriel, Rosalind L Smyth, Munir Pirmohamed

**Affiliations:** a Department of Women's and Children's Health, University of Liverpool, Liverpool, UK; b Wolfson Centre for Personalised Medicine, Medical Research Council (MRC) Centre for Drug Safety Science, Department of Molecular and Clinical Pharmacology, University of Liverpool, Liverpool, UK; c Department of Biostatistics, University of Liverpool, Liverpool, UK; d National Institute for Health Research (NIHR) Alder Hey Clinical Research Facility, Alder Hey Children's Hospital, Liverpool, UK; e Department of Endocrinology, Alder Hey Children's Hospital, Liverpool, UK; f Department of Respiratory Medicine, Alder Hey Children's Hospital, Liverpool, UK; g British Heart Foundation (BHF) Centre for Cardiovascular Science, University of Edinburgh, Edinburgh, UK; h MRC Integrated Epidemiology Unit at the University of Bristol, Bristol, UK; i Institute of Genetic Medicine, Newcastle University, Newcastle upon Tyne, UK; j Great Ormond Street Institute of Child Health, University College London, London, UK

## Abstract

**Background:**

A serious adverse effect of corticosteroid therapy is adrenal suppression. Our aim was to identify genetic variants affecting susceptibility to corticosteroid-induced adrenal suppression.

**Methods:**

We enrolled children with asthma who used inhaled corticosteroids as part of their treatment from 25 sites across the UK (discovery cohort), as part of the Pharmacogenetics of Adrenal Suppression with Inhaled Steroids (PASS) study. We included two validation cohorts, one comprising children with asthma (PASS study) and the other consisting of adults with chronic obstructive pulmonary disorder (COPD) who were recruited from two UK centres for the Pharmacogenomics of Adrenal Suppression in COPD (PASIC) study. Participants underwent a low-dose short synacthen test. Adrenal suppression was defined as peak cortisol less than 350 nmol/L (in children) and less than 500 nmol/L (in adults). A case-control genome-wide association study was done with the control subset augmented by Wellcome Trust Case Control Consortium 2 (WTCCC2) participants. Single nucleotide polymorphisms (SNPs) that fulfilled criteria to be advanced to replication were tested by a random-effects inverse variance meta-analysis. This report presents the primary analysis. The PASS study is registered in the European Genome-phenome Archive (EGA). The PASS study is complete whereas the PASIC study is ongoing.

**Findings:**

Between November, 2008, and September, 2011, 499 children were enrolled to the discovery cohort. Between October, 2011, and December, 2012, 81 children were enrolled to the paediatric validation cohort, and from February, 2010, to June, 2015, 78 adults were enrolled to the adult validation cohort. Adrenal suppression was present in 35 (7%) children in the discovery cohort and six (7%) children and 17 (22%) adults in the validation cohorts. In the discovery cohort, 40 SNPs were found to be associated with adrenal suppression (genome-wide significance p<1 × 10^−6^), including an intronic SNP within the *PDGFD* gene locus (rs591118; odds ratio [OR] 7·32, 95% CI 3·15–16·99; p=5·8 × 10^−8^). This finding for rs591118 was validated successfully in both the paediatric asthma (OR 3·86, 95% CI 1·19–12·50; p=0·02) and adult COPD (2·41, 1·10–5·28; p=0·03) cohorts. The proportions of patients with adrenal suppression by rs591118 genotype were six (3%) of 214 patients with the GG genotype, 15 (6%) of 244 with the AG genotype, and 22 (25%) of 87 with the AA genotype. Meta-analysis of the paediatric cohorts (discovery and validation) and all three cohorts showed genome-wide significance of rs591118 (respectively, OR 5·89, 95% CI 2·97–11·68; p=4·3 × 10^−9^; and 4·05, 2·00–8·21; p=3·5 × 10^−10^).

**Interpretation:**

Our findings suggest that genetic variation in the *PDGFD* gene locus increases the risk of adrenal suppression in children and adults who use corticosteroids to treat asthma and COPD, respectively.

**Funding:**

Department of Health Chair in Pharmacogenetics.

## Introduction

Inhaled corticosteroids (ICS) are recommended for adults and children with asthma and for chronic obstructive pulmonary disease (COPD).^1^, ^2^, ^3^, ^4^ Although ICS are generally well tolerated and have fewer systemic adverse effects than do oral corticosteroids,^5^ some patients can still develop systemic adverse effects. Adrenal suppression is a clinically important adverse effect, particularly in children with asthma, in whom the diagnosis of adrenal suppression can be challenging because presentation can range from asymptomatic biochemical changes to non-specific lethargy to florid adrenal crisis and death.

Several tests are available to assess adrenal function. The low-dose (1 μg) short synacthen test is the most widely used and correlates with the insulin tolerance test in adults.^6^ In children, the threshold for diagnosing adrenal suppression with the low-dose short synacthen test has been based on adult reference values (peak cortisol <500 nmol/L), but data suggest that, in patients treated with ICS, a threshold of peak cortisol less than 350 nmol/L might be more appropriate to identify clinically significant adrenal impairment.^7^

**Research in context**

**Evidence before this study**

We searched PubMed up to Jan 1, 2018, with the terms (“Adrenal suppression” OR “Adrenal insufficiency”) AND “corticosteroid” AND (“asthma” OR “Chronic Obstructive Pulmonary Disease” OR “COPD”) AND (“Pharmacogenetics” OR “Pharmacogenomics” OR “polymorphism”), for studies investigating any association between host genetic factors and susceptibility to adrenal suppression. We found no peer-reviewed publications investigating pharmacogenomic variants associated with peak cortisol in patients being treated with corticosteroids for asthma and COPD. However, several publications examined the relation between corticosteroid efficacy and genetic polymorphisms.

**Added value of this study**

As far as we know, this is the first pharmacogenomic study to investigate the association between a patient's genotype and corticosteroid-induced adrenal suppression. A polymorphism in the *PDGFD* gene locus was identified in a cohort of children with asthma in a genome-wide association study and found to be associated with adrenal suppression. This finding was validated in another paediatric asthma cohort and in a cohort of adults with chronic obstructive pulmonary disorder (COPD). A meta-analysis of the cohorts showed genome-wide significance.

**Implications of all the available evidence**

Our data support the idea of a link between interindividual variation in susceptibility to corticosteroid-induced adrenal suppression and a patient's genotype, potentially through variation in the *PDGFD* gene locus. This finding offers the potential to develop translational pathways to prevent corticosteroid-induced adrenal suppression, thereby improving the benefit–risk ratio of this important therapy.

Interindividual variation in susceptibility to adrenal suppression is striking. Baseline and peak cortisol levels in children with asthma treated with corticosteroids vary with age and sex.^8^ Although higher doses of corticosteroids are associated significantly with lower baseline and peak cortisol values,^8^, ^9^, ^10^ at a population level, the reduction in peak cortisol seen with increasing total dose of corticosteroids is small (<1 nmol/L per 200 μg/day).^8^ However, symptomatic adrenal suppression has been reported at doses as low as 200 μg of inhaled beclometasone per day,^11^ and one in three children with asthma using corticosteroids has a peak cortisol lower than 500 nmol/L.^8^ Adrenal suppression is also seen in adults with asthma and COPD treated with ICS.^5^ The reasons for the interindividual variability in both adults and children remain unclear, because clinical factors only account for a small proportion of the variance.^8^

Previous pharmacogenomic studies in patients with asthma using corticosteroids have focused on efficacy.^12^ As far as we know, no studies examining the pharmacogenomics of corticosteroid-induced adrenal suppression have been reported. The aim of the Pharmacogenetics of Adrenal Suppression with Inhaled Steroids (PASS) study was to undertake a pharmacogenomic assessment of factors predisposing to corticosteroid-induced adrenal suppression among children with asthma using ICS as part of their treatment. Validation was undertaken in both a paediatric asthma cohort (enrolled to the PASS study) and an adult COPD cohort (enrolled to the Pharmacogenomics of Adrenal Suppression in COPD [PASIC] study).

## Methods

### Participants

We recruited participants to the PASS study from 25 sites across the UK. We included children aged 5–18 years with asthma using ICS as part of their treatment (discovery cohort) and later recruited more children to a validation cohort. Full eligibility criteria have been published previously.^7^ Study participants in all paediatric cohorts were recruited either prospectively (if a low-dose short synacthen test had not yet been undertaken) or retrospectively (if the low-dose short synacthen test had already been done). The appendix presents further details of the recruitment strategy used for the PASS study, including the sample size calculation to set the recruitment target. We used single nucleotide polymorphism (SNP) array data from the Wellcome Trust Case Control Consortium 2 (WTCCC2) cohort as population control data for the genome-wide association case-control analysis.

We included a second validation cohort, which comprised adults aged 18 years or older with COPD and using ICS who were recruited from two sites in the UK for the PASIC study. Full eligibility criteria are included in the appendix. All adult participants were recruited to the study at the time the analysis of the PASS discovery cohort was undertaken.

The PASS study received ethics approval from Liverpool Paediatric Research Ethics Committee. The PASIC study received ethics approval from North West 2 Research Ethics Committee (Liverpool Central). All participants gave written informed consent.

### Procedures

To test adrenal suppression, we did the low-dose short synacthen test procedure as described previously.^8^ The appendix contains full details for all low-dose short synacthen tests and their interpretation. We defined adrenal suppression with cutoffs for peak cortisol of less than 500 nmol/L (in adults) and less than 350 nmol/L (in children).

For the genotype analysis, we obtained either whole-blood samples (maximum of 10 mL in EDTA tubes) or saliva samples (2 mL) for DNA extraction. Extraction methods are detailed in the appendix. We did genotype analysis of DNA samples from children in the discovery cohort at Edinburgh Genomics (The Roslin Institute, University of Edinburgh, Edinburgh, UK) on the Illumina Human OmniExpressExome-8v1 BeadChip using the Infinium HD Super assay (Illumina, San Diego, CA, USA). All data from the discovery cohort will be made available via the European Genome-phenome Archive (EGA). We did genotype analysis of samples from the paediatric asthma and adult COPD validation cohorts using Taqman allelic discrimination assay (Thermo Fisher Scientific, Paisley, UK). WTCCC2 controls underwent genotype analysis on a custom Illumina Infinium chip. The appendix contains further details on genotyping, quality control, and imputation.

### Statistical analysis

The appendix contains full details of statistical analyses. We did genome-wide case-control analyses with cutoffs for peak cortisol from the low-dose short synacthen test of less than 500 nmol/L and less than 350 nmol/L. To test for associations between every SNP in turn and case-control status, we fitted a logistic regression model in SNPtest version 2.4.1 (University of Oxford, Oxford, UK), assuming an additive genetic model. We also adjusted for population substructure (appendix). We calculated Q-Q plots of phenotypes to test for genomic inflation, with λ values (ratio of median of observed distribution to median of expected distribution) indicating genomic inflation.^13^ We identified significant SNPs using Manhattan plots (appendix). We did secondary analyses within the discovery cohort to test for association with peak and baseline cortisol levels as continuous phenotypes. We used LocusZoom version 0.4.8 (University of Michigan, Ann Arbor, MI, USA) to visualise genome-wide association analyses.

We did two meta-analyses of SNPs selected for validation (appendix) in meta-analysis helper (METAL; University of Michigan, Ann Arbor, MI, USA).^14^ Meta-analyses were done to determine the true effect sizes of polymorphisms more accurately. The first meta-analysis was done for the two paediatric cohorts using the peak cortisol less than 350 nmol/L phenotype, and the second was for all three cohorts combined using peak cortisol less than 500 nmol/L phenotype.

We compared our data with those from the CORtisol NETwork (CORNET) consortium genome-wide association meta-analysis^15^ to ensure that the polymorphisms identified were not associated with variation in cortisol in patients not exposed to corticosteroids. The association between rs591118 and morning plasma cortisol was investigated in 12 studies that participated in the discovery and replication stages of CORNET and in four additional studies that joined the CORNET consortium later. Full details of these datasets are shown in the appendix. We used MAGENTA version 2.4 (Broad Institute, MA, USA)^16^ to scrutinise the results from all phenotypes to identify any enrichment of functional and biological pathways (appendix).

### Role of the funding source

The funding source had no role in study design, data collection, data analysis, data interpretation, or writing of the report. ALJ, BF, DBH, and MPi had access to raw data. The corresponding author had full access to all the study data and had final responsibility for the decision to submit for publication.

## Results

Between November, 2008, and September, 2011, 499 children were enrolled to the discovery cohort. Characteristics of these patients, including those categorised as showing adrenal suppression, are shown in table 1. 92 (18%) children failed genotype quality control, of whom 19 did not meet sample call-rate criteria, five failed the sex check, nine did not meet identity-by-descent criteria, and 59 were excluded as population outliers after principal component analysis; none failed the heterozygosity rate check (appendix). Therefore, 407 were included in the genome-wide association study. In total, 951 117 SNPs were genotyped; 654 246 SNPs passed the predefined genotyping quality-control criteria, of which 430 492 overlapped with WTCCC2 SNPs.

**Table 1:** Demographics of discovery and validation cohorts

		**PASS discovery cohort (n=499)**	**PASS paediatric validation cohort (n=81)**	**PASIC adult validation cohort (n=78)**
Sex				
	Female	209 (42%)	36 (44%)	37 (47%)
	Male	290 (58%)	45 (56%)	41 (53%)
Age (years)		11·6 (3·3)	14·6 (4·9)	65·8 (15·0)
Weight (kg)		43 (18)	44 (19)	75 (19)
Total daily oral dose (μg beclometasone equivalent)		6915 (17 014)	10 638 (30 127)	12 878 (7163)
Regular ICS		499 (100%)	81 (100%)	78 (100%)
Rescue corticosteroid use		264 (53%)	42 (53%)	78 (100%)
Regular oral corticosteroid		50 (10%)	7 (9%)	0 (0%)
Biochemical adrenal insufficiency (peak cortisol)				
	<350 nmol/L	35 (7%)	6 (8%)	NA
	<500 nmol/L	175 (35%)	32 (40%)	17 (22%)

Data are number (%) or mean (SD). ICS=inhaled corticosteroids. NA=not applicable.

**Figure 1:**
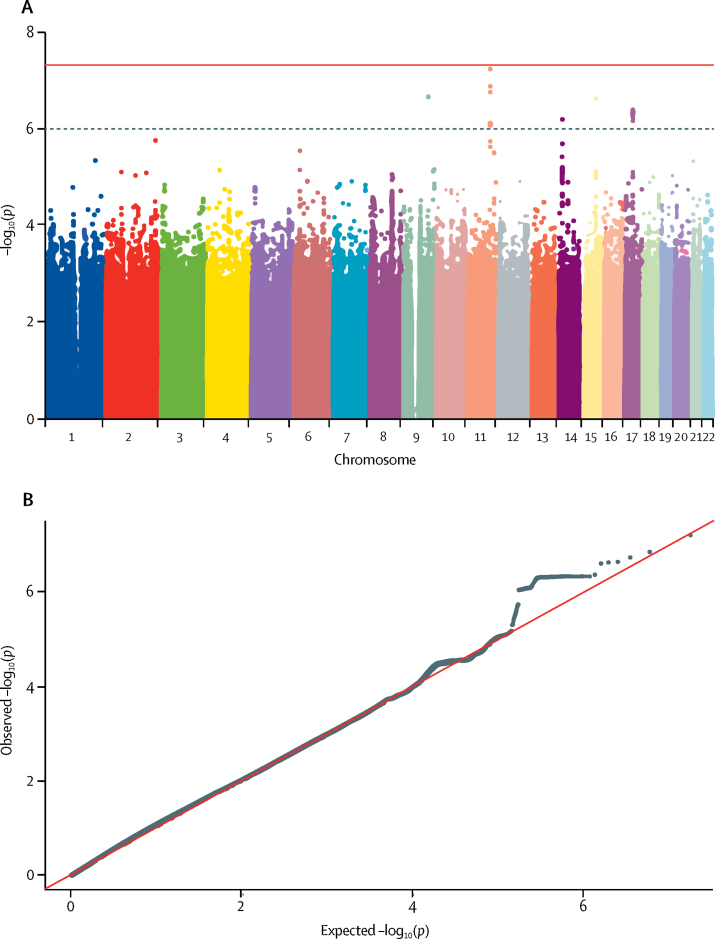
Manhattan (A) and Q-Q (B) plots of genome-wide data relating to dichotomous outcome of peak cortisol less than 350 nmol/L Data obtained by undertaking a low-dose short synacthen test in all participants. (A) Blue line represents the notional statistical significance threshold; red line represents the genome-wide statistical significance threshold. (B) Red diagonal line is the unity line indicating the quintiles of the p values come from the same distribution.

**Figure 2:**
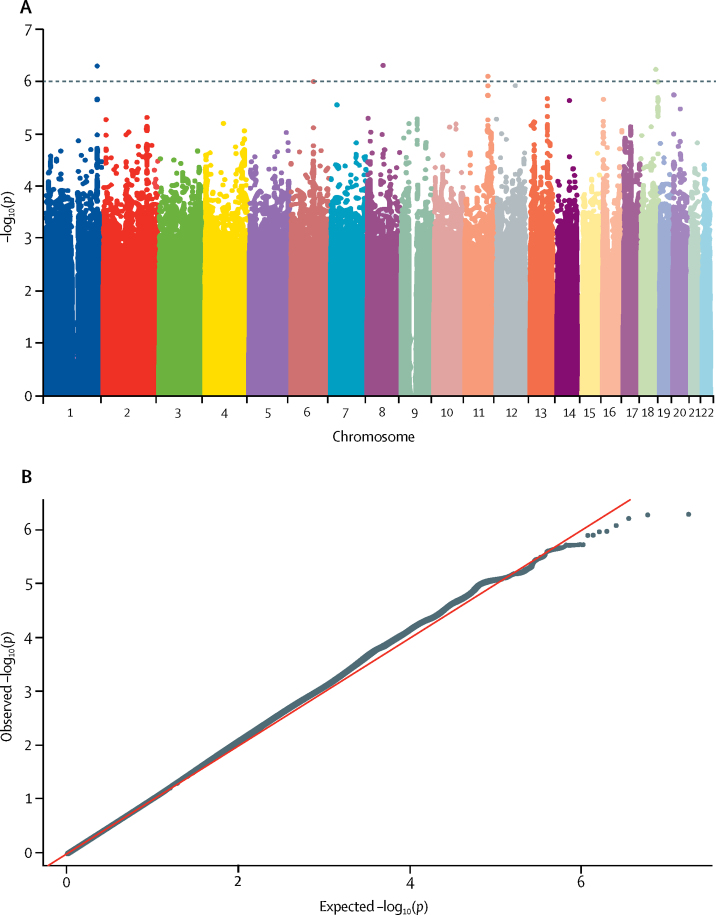
Manhattan (A) and Q-Q (B) plots of genome-wide data relating to dichotomous outcome of peak cortisol less than 500 nmol/L Data obtained by undertaking a low-dose short synacthen test in all participants. (A) Blue line represents the notional statistical significance threshold. (B) Red diagonal line is the unity line indicating the quintiles of the p values come from the same distribution.

**Figure 3:**
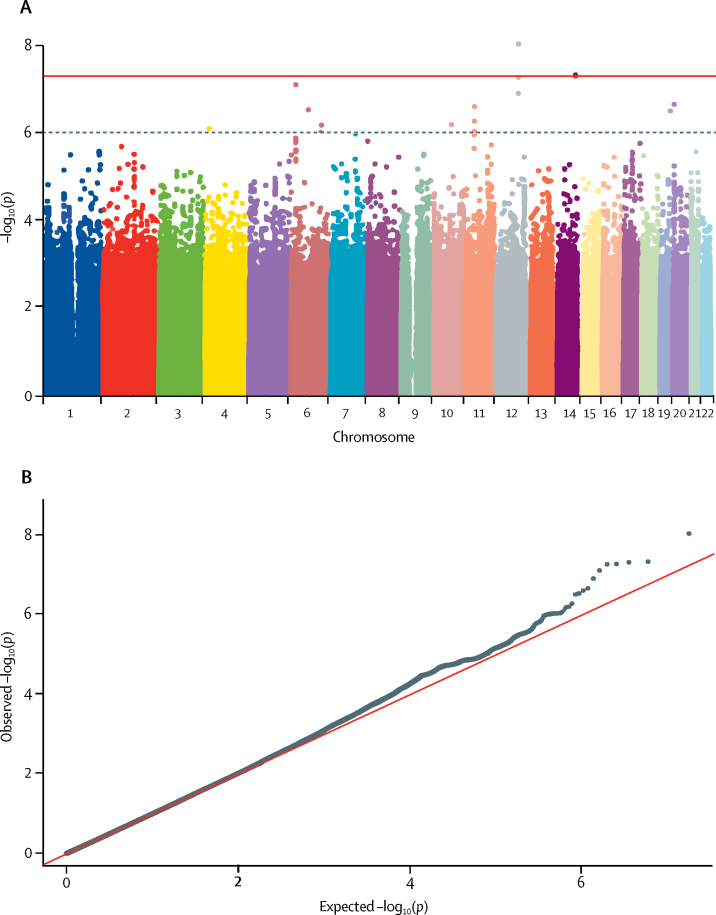
Manhattan (A) and Q-Q (B) plots of genome-wide data relating to continuous outcome of peak cortisol levels Data obtained by undertaking a low-dose short synacthen test in all participants. (A) Blue line represents the notional statistical significance threshold; red line represents the genome-wide statistical significance threshold. (B) Red diagonal line is the unity line indicating the quintiles of the p values come from the same distribution.

**Figure 4:**
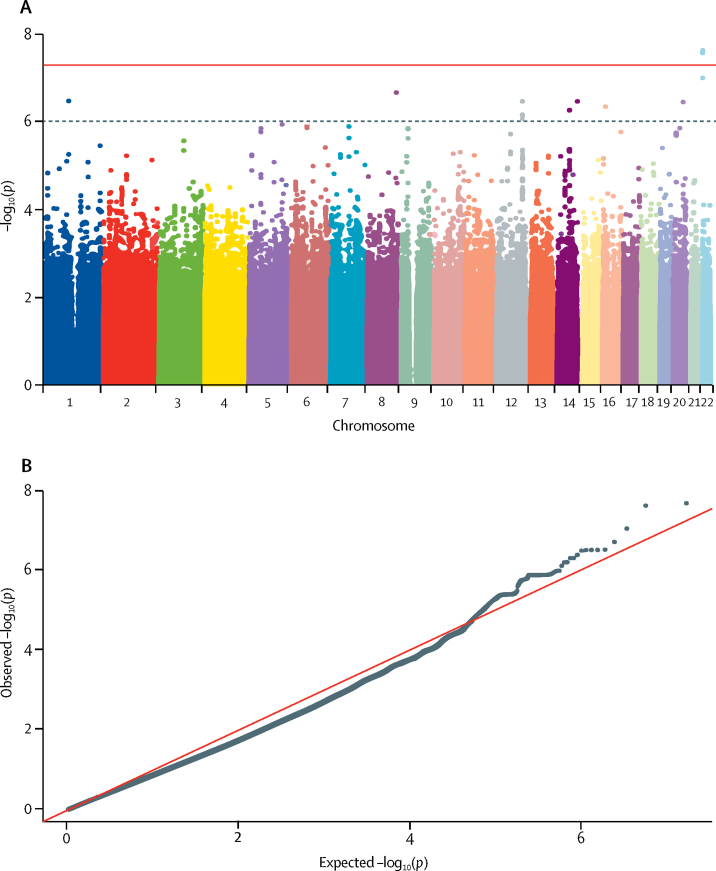
Manhattan (A) and Q-Q (B) plots of genome-wide data relating to continuous outcome of baseline (log-transformed) cortisol levels Data obtained by undertaking a low-dose short synacthen test in all participants. (A) Blue line represents the notional statistical significance threshold; red line represents the genome-wide statistical significance threshold. (B) Red diagonal line is the unity line indicating the quintiles of the p values come from the same distribution.

Figure 1, Figure 2, Figure 3, Figure 4 display Q-Q plots for the four phenotypes—namely, peak cortisol less than 350 nmol/L (figure 1B), peak cortisol less than 500 nmol/L (figure 2B), continuous peak cortisol (figure 3B), and continuous baseline cortisol (figure 4B). No genomic inflation was evident in the genome-wide association study for the phenotypes peak cortisol less than 500 nmol/L (λ=0·99), peak cortisol less than 350 nmol/L (λ=1·03), or continuous peak cortisol (λ=0·99). However, genomic inflation was noted for the continuous baseline cortisol phenotype (λ=1·22). Because of the rigorous quality-control procedures applied to the dataset, the common causes of genomic inflation—eg, undetected sample duplications, unknown familial relationships, a poorly calibrated test statistic, systematic technical bias, or gross population stratification—were eliminated. Therefore, because the cause of genomic inflation was unknown, the p values in the analysis of the baseline phenotype were adjusted for genomic inflation.

Figure 1, Figure 2, Figure 3, Figure 4 also show Manhattan plots for the four phenotypes. 51 SNPs were identified initially as notionally statistically significant (p<1 × 10^−6^) for the phenotype peak cortisol less than 350 nmol/L (figure 1A), with four SNPs identified for the phenotype peak cortisol less than 500 nmol/L (figure 2A), 23 identified for the phenotype continuous peak cortisol (figure 3A), and 16 identified for the phenotype baseline cortisol (figure 4A).

SNPs that were notionally statistically significant (p<1 × 10^−6^) in the analyses of association with each phenotype in turn are shown in table 2, together with the genetic region and information on whether the SNP was genotyped or imputed. In total, 60 SNPs were identified in 18 genes across the phenotypes. SNPs in *PDGFD* were the only variants associated with more than one phenotype (peak cortisol <500 nmol/L and <350 nmol/L). The ranking of p-value associations was undisturbed for the top 20 SNPs when the analysis was repeated after excluding the WTCCC2 control group (appendix).

**Table 2:** Genes containing SNPs notionally statistically significant (p<1 × 10^−6^) identified in the discovery cohort

	**Single nucleotide polymorphisms identified**	**Lowest p value in discovery cohort**
**Peak cortisol <500 nmol/L**		
*GJA8* (chromosome1)	rs201161441, rs6657114,*†‡ rs6671502	1·69 × 10^−22^
*TRPA1* (chromosome 8)	rs75470088†	5·04 × 10^−7^
*PDGFD* (chromosome 11)	rs7116655†	8·14 × 10^−7^
**Peak cortisol <350 nmol/L**		
*PDGFD* (chromosome 11)	rs361283, rs361284, rs590216,* rs603781,* rs591118,† rs589796, rs2515080, rs684212, rs517401, rs671851, rs2515083, rs620426, rs619954, rs574494, rs619114, rs618648, rs5794293, rs623031	5·80 × 10^−8^
*KRT8P9* (chromosome 15)	rs111566682†	2·32 × 10^−7^
*PSMD3* (chromosome 17)	rs9912981, rs3859188, rs71355433, rs7222556, rs9916279,*† rs8080546, rs11654706, rs11078932, rs58212353, rs2012	4·37 × 10^−7^
*CSF3* (chromosome 17)	rs2827†	4·48 × 10^−7^
*MED24* (chromosome 17)	rs11555254,* rs2302778, rs7503939, rs17850739,*† rs72834789	4·02 × 10^−7^
**Peak continuous cortisol**		
*LRP1B* (chromosome 2)	rs142320277†	5·23 × 10^−8^
*GBA3* (chromosome 4)	rs111863753†	7·75 × 10^−7^
*HMGN3* (chromosome 6)	rs13220233†	2·88 × 10^−7^
*PDE7B* (chromosome 6)	rs149647891†	6·43 × 10^−7^
*SCGN* (chromosome 6)	rs5875060†	7·62 × 10^−8^
*ANKS1B* (chromosome 12)	rs191087489, rs143638033, rs142161979†	8·98 × 10^−9^
*ELSPBP1* (chromosome 19)	rs137939366†	3·05 × 10^−7^
**Baseline continuous cortisol**		
*NOS1* (chromosome 12)	rs12815584, rs77562913, rs76830467,† rs75992652, rs34406980, rs150941488	3·00 × 10^−7^
*IGH* (chromosome 14)	rs201541519†	3·02 × 10^−7^
*SLC2A10* (chromosome 20)	rs117420762†	3·12 × 10^−7^
*BCL2L13* (chromosome 22)	rs149352662, rs189673743, rs140179402†	2·00 × 10^−8^

* SNPs identified by genotype rather than imputation.

† Corresponds to the lowest p value.

‡ Probable artifact after review of Manhattan plots.

The LocusZoom plot of regions on chromosome 11 where *PDGFD* is located (appendix) suggested a true signal. The interaction analysis between the phenotype peak cortisol less than 350 nmol/L and rs591118 SNP was not significant (p=0·24) indicating that the interaction between drug dose and SNP is unlikely to be associated with outcome. A similar linkage disequilibrium structure was noted in the LocusZoom plot of the *TRPA1* gene located in chromosome 8. LocusZoom plots for genes other than *PDGFD* and *TRPA1* were not supportive of a true signal in terms of the linkage disequilibrium structures seen.

In view of the association identified with *PDGFD* SNPs and evidence of biological plausibility (appendix), this gene was selected for investigation in the validation cohorts. Further analysis of this region of *PDGFD* and the phenotype peak cortisol less than 350 nmol/L, without the WTCCC2 controls and adjusted for significant clinical factors, showed that the signal was robust (appendix). The individual *PDGFD* SNP selected to be genotyped in the validation cohorts was rs591118, which had a significant association with the peak cortisol less than 350 nmol/L phenotype (odds ratio [OR] 7·32, 95% CI 3·15–16·99; p=5·8 × 10^−8^).

Between October, 2011, and December, 2012, 81 children with asthma were recruited to a paediatric asthma validation cohort. A further validation cohort of adults with COPD were recruited between February, 2010, and June, 2015. Characteristics of these two validation cohorts are shown in table 1. Validation was done initially in the paediatric asthma cohort. Analyses of rs591118 and case-control status with the phenotypes peak cortisol less than 500 nmol/L (OR 2·12, 95% CI 1·03–4·37; p=0·04) and less than 350 nmol/L (3·86, 1·19–12·50; p=0·02) were significant. In the adult validation cohort, analysis of the association between rs591118 and the prespecified phenotype peak cortisol less than 500 nmol/L was significant (OR 2·41, 95% CI 1·10–5·28; p=0·03). Peak cortisol responses were generally lower in individuals who had the GG genotype at rs591118 (appendix). A peak cortisol response of either less than 350 nmol/L (children) or less than 500 nmol/L (adults) was achieved by six (3%) of 214 participants with the GG genotype, 15 (6%) of 244 with the AG genotype, and 22 (25%) of 87 with the AA genotype.

**Figure 5:**
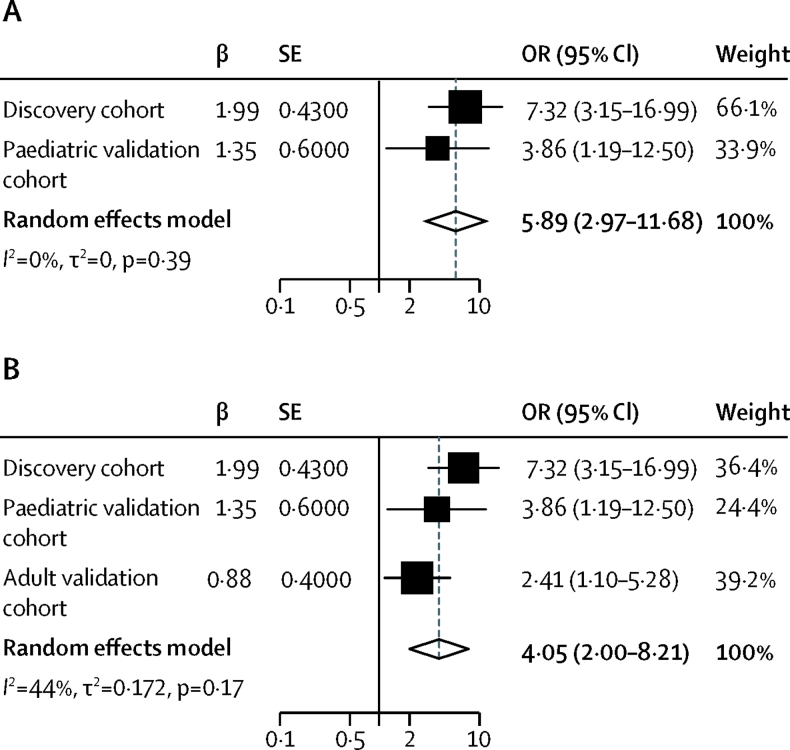
Meta-analysis of (A) all children with asthma treated with corticosteroids and (B) all children with asthma and adults with COPD OR=odds ratio.

By comparison with CORNET consortium data, which comprised the extended CORNET cohort of 24 467 individuals of European ancestry, no evidence was found that genetic variation at rs591118 was associated with morning plasma cortisol (mean difference per A allele 0·002 SD, SE 0·008; p=0·80). 16 pathways were included in the pathway analysis that contained the *PDGFD* gene. Two pathways were identified that were associated with a false-discovery rate-corrected p value less than 0·05 (appendix). Meta-analysis of the two paediatric cohorts was significant for the phenotype peak cortisol less than 350 nmol/L (OR 5·89, 95% CI 2·97–11·68; p=4·3 × 10^−9^; figure 5A), and meta-analysis of all three cohorts for the peak cortisol less than 500 nmol/L phenotype was also significant (OR 4·05, 95% CI 2·00–8·21; p=3·5 × 10^−10^; figure 5B).

## Discussion

The findings of our genome-wide association study, examining genetic factors associated with corticosteroid-induced adrenal suppression in children with asthma treated with predominantly ICS, showed that common variants in the *PDGFD* gene (eg, rs591118) were associated with the development of adrenal suppression. The findings were validated in another group of children with asthma and in a group of adults with COPD and the rs591118 variant was found to have genome-wide significance in both. The validation in the adult cohort is especially remarkable since these people were suffering from a different complex disease, had multiple comorbidities, and were on multiple medications, further reinforcing our novel finding. Heterogeneity was higher (44%) with the meta-analysis of the COPD cohort compared with the asthma cohort (0%), which could reflect heterogeneity of the underlying disease.

The SNP rs591118 is an A→G substitution in an intronic region of the *PDGFD* gene. Analysis of the Genotype-Tissue Expression Portal^17^ shows that this variant does not alter PDGFD expression, but does have a score of 3a on RegulomeDB,^18^ suggesting that rs591118 might affect transcription factor binding. However, further fine-mapping will be needed to identify definitively the causal variant. A search of the National Human Genome Research Institute Genome-Wide Association Study catalogue on Aug 14, 2017, showed that this particular SNP has not been associated with any disease or trait. The only associations with other variants within *PDGFD* of genome-wide significance (p<5 × 10^−8^) have been reported with coronary artery disease and myocardial infarction (rs974819, rs2128739, and rs2019090), with linkage disequilibrium 0·03 or less.^19^, ^20^

Platelet-derived growth factors (PDGFs) direct the migration, differentiation, and function of various specialised mesenchymal cell types.^21^ PDGF receptors are required for development of steroid-producing cells in many organs, including the testes, ovaries, and adrenal cortex.^22^ Moreover, PDGFD is highly expressed in human adrenal gland,^23^, ^24^ unlike PDGFA, PDGFB, and PDGFC, and the expression of PDGFD correlates negatively with cortisol secretion in adrenocortical adenomas.^25^ However, our analysis does not suggest that the rs591118 SNP is a marker of constitutional variation in cortisol production because it was not associated with morning plasma cortisol levels among steroid-naive individuals in the CORNET consortium.^15^

We considered whether *PDGFD* genotype might affect asthma or COPD severity, and hence corticosteroid dose used in the patients. Although *PDGFB* is associated with alterations in airway remodelling in asthma,^26^ a link to *PDGFD* has not been made. Several polymorphisms have been associated with development and severity of asthma and COPD,^27^, ^28^, ^29^ but none are in *PDGFD*. Also, polymorphisms in *PDGFD* have not been identified as affecting efficacy of corticosteroid treatment.^12^ Taken together, these data suggest that patients with adrenal suppression attributable to *PDGFD* variants do not have more severe asthma or reduced response to ICS. Genetic variation in the *PDGFD* gene might determine responsiveness of the adrenal gland to corticotropin rather than susceptibility to suppression of corticotropin production, but either of these mechanisms is likely to be important in determining susceptibility to clinically significant corticosteroid-induced adrenal suppression.

Although we have focused on the *PDGFD* locus, and identified the potential biological reasons for the involvement of PDGFD in corticosteroid-induced adrenal suppression, it is possible that the actual gene predisposing to this phenotype might be located distantly from rs591118. Therefore, laboratory and human experimental studies will be needed to understand whether variation at the *PDGFD* locus affects adrenal function.

What is the clinical significance of our finding? The minor allele frequency for rs591118 is 0·44,^30^ and the current prevalence of childhood asthma is 11%,^31^ meaning that roughly 2% of all children (276 000 in the UK and 1·58 million in the USA) will have both asthma and be homozygous for rs591118. For COPD, 1·2 million and 11 million adults in the UK and USA, respectively, have the disease, with about 232 000 in the UK and 2 130 000 in the USA homozygous for the SNP. Children homozygous for the minor allele of rs591118 who have asthma and use ICS are nearly six times more likely to develop adrenal suppression than are children who have asthma and use ICS who are homozygous wild-type. Similarly, adults homozygous for the minor allele of rs591118 who have COPD and use high-dose ICS are four times more likely to develop adrenal suppression than are adults who have COPD and use ICS who are homozygous wild-type. Corticosteroids are used to treat many disorders, not merely asthma and COPD. Future studies should, thus, assess other populations of patients treated with corticosteroids and investigate whether this SNP is associated with other corticosteroid adverse effects.

The possible clinical relevance of our study finding might lie in identifying individuals who would benefit from use of alternative treatments to ICS—eg, in paediatric asthma, leukotriene receptor antagonists. Our calculations show that 27 children with asthma, or 12 adults with COPD, would need to be tested and put on an alternative therapy to avoid one case of adrenal suppression (appendix). However, this approach cannot be advocated without further studies, which might include a biomarker-stratified randomised controlled trial. An alternative scenario (if ICS are still needed) would be to identify patients who need more intensive monitoring of adrenal function. Whether both of these clinical interventions would be cost-effective is unknown, and would need to be built into future study designs.

A limitation of our study is the small sample size used for the genome-wide association study when compared with studies using genome-wide association to investigate individual susceptibility to developing a complex disease. However, significant associations between genetic polymorphisms and serious adverse drug reactions that have led to changes in practice have previously been identified from small cohorts.^32^ We identified other genetic loci that did not reach genome-wide significance, including *TRPA1* (which we did not assess further), and a larger sample size might identify additional loci. Because the low-dose short synacthen test is a painful intervention, requiring cannulation, we restricted paediatric recruitment to children in whom there was clinical suspicion of adrenal suppression. These inclusion criteria might mean we enriched our study population artificially, because clinicians will have selected children they perceived to be most at risk, but this approach is in keeping with clinical risk stratification. Finally, the small sample size may have affected the calculation for number needed to treat.

In conclusion, we have shown that a common variation in the *PDGFD* locus is associated with an increased risk of adrenal suppression. Our finding is highly novel, and its importance is emphasised by its validation in a multimorbid adult population with COPD. Further work is needed not only in larger patient cohorts to characterise the effect size of rs591118 with greater accuracy but also to define the functional mechanisms by which *PDGFD* is involved in corticosteroid-induced adrenal suppression.

For the **EGA** see https://ega-archive.org
