# Supplementary material for: Susceptibility to corticosteroid-induced adrenal suppression: a genome-wide association study
Source: Lancet Respir Med. 2018 Jun;6(6):442–50. doi: 10.1016/S2213-2600(18)30058-4 (PMC5971210; doi:10.1016/S2213-2600(18)30058-4)
Supplement: Supplementary appendix [file mmc1.pdf]

THE LANCET
Respiratory Medicine

Supplementary appendix

This appendix formed part of the original submission and has been peer reviewed. We post it as supplied by the authors.

Supplement to: Hawcutt D B, Francis B, Carr D F, et al. Susceptibility to corticosteroid-induced adrenal suppression: a genome-wide association study. *Lancet Respir Med* 2018; published online March 15. http://dx.doi.org/10.1016/S2213-2600(18)30058-4.

Genome-wide association study of susceptibility to corticosteroid induced adrenal suppression.

Supplementary Data Section

Daniel B. Hawcutt,[1,2] MD, Ben Francis[3] PhD, Daniel F. Carr [2] PhD, Andrea L Jorgensen[3] PhD, Peng Yin[3] PhD, Naomi Rogers[1] BSc (Hons), Natalie O'Hara[2] B.Sc (Hons), Eunice J. Zhang[2] PhD, Katarzyna M. Bloch[2] PhD, Amitava Ganguli[2] MRCP, Ben Thompson[2] MB ChB, Laurence McEvoy[2] BSc (Hons) , Professor Matthew Peak[4] PhD, Andrew A Crawford[5, 6] PhD, Professor Brian R Walker[5,7] MD, Joanne C Blair[8] MD, Jonathan Couriel[9] MA, Professor Rosalind Smyth[10] FMedSci, Professor Munir Pirmohamed[2] FMedSci

1: Department of Women's and Children's Health, University of Liverpool, Liverpool, UK

2: Wolfson Centre for Personalised Medicine, MRC Centre for Drug Safety Science, Department of Molecular and Clinical Pharmacology, University of Liverpool, Liverpool, UK

3: Department of Biostatistics, University of Liverpool, Liverpool, UK

4: NIHR Alder Hey Clinical Research Facility, Alder Hey Children's Hospital, Liverpool, UK

5: BHF Centre for Cardiovascular Science, University of Edinburgh, Edinburgh, UK

6: MRC Integrated Epidemiology Unit as the University of Bristol, Bristol, UK

7: Institute for Genetic Medicine, Newcastle University, Newcastle upon Tyne, UK

8: Department of Endocrinology, Alder Hey Children's Hospital, Liverpool, UK

9: Department of Respiratory Medicine, Alder Hey Children's Hospital, Liverpool, UK

10: Great Ormond Street Institute of Child Health, University College London, UK

Methods

PASS Study

Recruitment

The PASS study recruited children in two stages, firstly for a discovery cohort, and subsequently for a validation cohort. The validation cohort was specifically recruited for this purpose. There were no differences in the protocol between the two phases of recruitment.

Consent process

Participants and families were identified by hospital paediatricians. Information was given to the family and consent obtained from the parent/guardian (or participant if ≥16 years) after at least 24 hours consideration. Assent was also sought from older participants (age 12-15 years, judged on a case by case basis). Participants judged able to give assent, but who declined, were excluded.

Design of the LDSST

We based the sampling regimen for LDSST on that developed by Paton et al [1]. In that study, LDSST consisted of a dose of 500ng/1.73m^2 and sampling at 0, 15, 20, 25, 30 and 35 minutes. Raw data from that audit [1] were reviewed and measurements from four of the six time points (0, 15, 25, 35 minutes) were able to consistently distinguish between normal and abnormal responses (18).   A minority of patients included in PASS were recruited having already undergone a LDSST within the last 2 years using the local protocol at the recruiting institution.

Statistical analysis

For the preliminary study of demographic and clinical factors, analyses were undertaken in SAS v.9.2 (Cary, NC, US). For times when multiple comparisons were carried out, a Bonferroni correction was used and a significant p-value assumed to be <0.005. Full details of the statistical methodology are included in the online supplementary data section of the Hawcutt et al. (2015)[2] paper. Analyses undertaken to investigate the growth observed in the population (height-for-age z score), and the

relationship between cumulative corticosteroid dose and height-for-age z score, are all detailed in the supplementary data section of the Hawcutt et al. (2015)[2] paper.

Calculating corticosteroid cumulative dose

Doses of corticosteroid taken by participants were expressed as micrograms/day of beclomethasone diproprionate equivalent [3]. Dose ratios of 1:1 (Beclomethasone Diproprionate CFC, Clenil Modulite, Budesonide) [4], 2:1 (Fluticasone, Mometasone, Qvar) [4] and 1:3.9 (Prednisolone) were used [5]. Patient's corticosteroid cumulative dose over the previous six months was then calculated in three different ways:

1) Mean daily inhaled/intranasal corticosteroid cumulative dose in six months prior to LDSST (cumulative dose of inhaled and intranasal corticosteroid medications, regardless of rescue therapies and excluding those on regular oral corticosteroids, expressed as a mean daily dose of beclomethasone diproprionate equivalent).
2) Number of courses of rescue oral corticosteroids required to treat exacerbations of asthma in the preceding 6 months before the LDSST.
3) Mean daily total corticosteroid cumulative dose in six months prior to LDSST (cumulative dose of inhaled, intranasal and regular oral corticosteroid medications as well as rescue courses, assuming a course of rescue corticosteroids is a three day course of oral prednisolone, dosed at 2 mg/kg per day (to a maximum dose of 40 mg), expressed as a mean daily dose of beclomethasone diproprionate equivalent).

All dose calculations assume 100% concordance and bioavailability (regardless of route of administration). Topical corticosteroid preparations applied to the skin were not included in the total corticosteroid dose calculations due to uncertainty about the dose received due to variations in both the surface area of the body affected and quantity of cream applied.

Adherence with corticosteroids in asthma has previously been assessed using prescription fill-refill data [6]. Repeat prescription data for the 6 months prior to the date of the LDSST were supplied by general practitioners (GP) of patients recruited from a single centre. Using the strength of inhaler prescribed, number of doses per inhaler (from manufacturers SPC) and prescribed dose, the minimum number of refills required by the patient in the 6 month period was calculated and

compared with the actual number of refills collected. For the statistically significant relationship(s), the effect of variation in the population adherence was calculated.

PASIC STUDY

Inclusion Criteria:

Patient willing to take part

COPD diagnosis by formal spirometry (spirometry result within the last 24 months)

Age over 18 to 80

Able to give Informed Consent

Regular Use of inhaled corticosteroids for over 12 months

High dose of inhaled corticosteroids as defined by > 500mcg Fluticasone or its equivalent dose of alternative ICS based on BTS guidelines[7] .

Exclusion Criteria:

History of anaphylaxis to synacthen

History of adrenal disease

On long term oral prednisolone or hydrocortisone or taken oral corticosteroid in the last 4 weeks

Patients taking Nasal steroids

Participating in another study.

Patients with a significant disease other than COPD; a significant disease is defined as a co-morbidity (respiratory and non-respiratory) which, in the opinion of the investigator, may

i) Put the patient at risk because of participation in the study,
ii) Influence the results of the study, or
iii) Cause concern regarding the patient's ability to participate in the study

This would include patients with a significant diagnosis of pulmonary fibrosis or bronchiectasis.

Patients with a current diagnosis of asthma

Patients with active malignancy

Known active tuberculosis.

Patients who regularly use daytime oxygen therapy for more than one hour per day and in the investigator's opinion will be unable to abstain from the use of oxygen therapy during clinic visits.

Pregnancy

Recruitment sites and recruitment leads

The following UK recruiting centres and Principal Investigators were involved in the PASS study:

Alder Hey Children's Hospital (Professor Rosalind Smyth), Arrowe Park Hospital (Dr David Lacy), Royal Manchester Children's Hospital (Dr Clare Murray), Whiston Hospital (Dr Laweh Amegavie), East Sussex Hospital (Dr Kanumakala), Southport and Ormskirk Hospital (Dr Gardner), North Manchester Hospital (Dr Dasgupta), Blackburn Hospital (Dr Robertson), Macclesfield Hospital (Dr Ho), Countess of Chester Hospital (Dr Steven Bearey), West Sussex Hospital (Dr Matthews/Dr Linney), Wigan Hospital (Dr Velmurugan), Leeds General Infirmary (Dr Lee), Warrington Hospital (Dr Nick Wild), Preston Hospital (Dr Mahmood), Barrow in Furness Hospital (Dr Olabi), Bradford Hospital (Dr Moya), Oldham Hospital (Dr Prakash), North Tees Hospital (Dr Tuladhar), Leighton Hospital (Dr Ellison), Tameside Hospital (Dr Levy), Wolverhampton Hospital (Dr Raynor), Sheffield Children's Hospital (Dr Wright), Doncaster Hospital (Dr Natarjan), Mid Yorkshire (Pinderfields) Hospital (Dr Jones), Huddersfield/Calderdale Hospital (Dr Garside), Queens Medical Centre Nottingham (Dr Bhatt), Stockport Hospital (Dr Cooper), Ayrshire Hospital (Dr Findlay/Dr Adams), Royal Cornwall Hospital (Dr Prendiville), Royal Hospital for Children, Glasgow (Dr Paton).

The following UK recruiting centres and Principal Investigators were involved in the PASIC study:

The Royal Liverpool & Broadgreen University Hospital NHS Trust (Dr Amitav Ganguli), Manchester Evaluation Unit: Wythenshawe Hospital, Manchester (Dr Dave Singh), The Royal Wolverhampton Hospitals NHS Trust: New Cross Hospital (Dr Lee Dowson), Medway NHS Foundation Trust: Medway Maritime Hospital (Dr Alisdair Stewart), The James Cook University Hospital (Dr Anur Guhan/ Dr

Ramamurthy Sathyamurthy/ Dr Mustafa), Lancashire Teaching Hospitals NHS Foundation Trust (Dr P Marsden), North Tees and Hartlepool NHS Trust (Dr Richard Harrison), Freeman Hospital (Dr Anthony de-Soyza), Countess of Chester Hospital (Dr Stephen Scott), Arrowe Park (Dr Nikki Stevenson), North Bristol NHS Trust (Prof Ann Millar), University Hospital North Durham (Dr Neil Munro), Princess Royal Hospital (Dr Moudgil), The York Hospital (Dr Rebecca Thomas), NHS Ayrshire and Arran (Dr Anur Guhan), University Hospitals of Morcambe Bay NHS Foundation Trust (Dr Tim Gatheral).

LDSST procedure and interpretation

For all prospectively recruited patients (PASS and all PASIC patients), the LDSST procedure was undertaken as follows: all tests were commenced before 11:00am and as close to 7:00am as possible. Participants were not fasted, but any corticosteroid medicine was withheld on the morning of the LDSST until test completion. For patients in the PASS cohort treated with alternate day oral corticosteroid, the LDSST was performed on the day a dose was due to be given, withholding it until completion of the LDSST.

An indwelling venous catheter was sited following the application of local anaesthetic cream. A blood sample was collected (time 0). One hundred and twenty five micrograms (0.5ml) Synacthen (Alliance, Chippenham, UK) 0.25mg/ml solution was added to 500ml 0.9%Nacl (final concentration of 250nanograms/ml) and agitated. For PASS, five hundred nanograms/1.73m^2 was administered as a bolus injection directly into the cannula and samples were collected 15, 25 and 35 minutes following Synacthen administration[8]. A Synacthen dose calculator was used at all sites to ensure consistency of dosing at different study locations. For PASIC, 1 microgram of Synacthen was used[9,10], with samples collected at 0, 30 and 60 minutes after injection with low dose Synacthen. For PASS and PASIC, all participating hospitals were required to be experienced in undertaking a LDSST, and have guidelines for management of anaphylaxis.

For retrospectively recruited patients (subset of PASS patients only), the LDSST was undertaken using the clinical protocol from the recruiting hospital. The dose of Synacthen used, timing of the LDSST and timing of sample collection were all recorded.

Identification of the correct threshold for diagnosis of adrenal suppression in children is difficult, as there are limited normative data. For paediatric populations, a peak cortisol of 350nmol/L has been shown to be the most sensitive and specific at determining adrenal insufficiency[8], although a threshold of 500nmol/L is still widely used clinically. For adults, 500nmol/L is the accepted

threshold. In a detailed review of symptomatic cases of adrenal insufficiency during ICS use in childhood asthma[8], there were no published cases in patients with peak cortisol concentrations >350nmol/L in either standard dose or low dose tests. For this study, we have therefore used <350nmol/L as our primary outcome for children within the PASS study populations, and <500nmol/L for the adults in the PASIC study.

All of the NHS trusts participating in this study analysed their own cortisol samples. All NHS laboratories subscribe to an external quality assurance (EQA) scheme, through the UK National Quality Assurance Scheme (UKNEQAS), to ensure consistency and robust analytical quality.

Sample size calculation

The target sample size for the primary analysis cohort was 500. To arrive at this estimate we considered two possible scenarios: (a) an odds ratio (OR) of 3 for association between a rare variant (minor allele frequency=5%) and the primary outcome; (b) an OR of 2 for association between a common variant (minor allele frequency=20%) and the primary outcome. A liberal type 1 error rate of 5% was assumed on the basis that validation cohorts would also be analysed to help eliminate false positives arising from the initial analyses. Assuming first of all prevalence of impaired adrenal response in children with asthma using inhaled steroid to be 17% (based on a the lower end of the range of previously published rates of adrenal suppression[2]) the power for scenario a) was calculated as 77% and the power for scenario b) was calculated as 75%. If the prevalence was 40% (the upper end of the range of published estimates[2]), the power increased to 91% for both scenarios. A prevalence of 20% would ensure power of at least 80% in both scenarios.

DNA Storage and Extraction

For all cohorts, patient samples for DNA were collected as whole blood (EDTA) or salivary samples (2ml saliva). DNA collection and extraction for saliva samples has been described previously[11]. EDTA blood samples were stored at -20°C and, following defrosting, genomic DNA was extracted using the Chemagen whole-blood DNA extraction kit on the Chemagic Magnetic Separation Module I according to the manufacturer's protocol (PerkinElmer chemagen Technologie GmbH, Baesweiler, Germany; www.chemagen.com).

Genotyping quality control (QC) and Imputation

Patients within the primary analysis cohort were excluded from association analyses if any of the following criteria were met: a) gender as determined by the “Sex Check” function within PLINK [12]

differed from that reported in the clinical data, b) the genotype call-rate was <90%, c) the pairwise identity by descent (IBD) statistic of relatedness was >0.1875 (patient with lowest call rate of the pair excluded); d) the heterozygosity rate is more than three standard deviations from the mean heterozygosity rate; or e) Principle component analysis (PCA) (using SNPRelate [13] in R v3.01) demonstrated that the individual's ethnicity did not cluster with the HapMap CEU (Utah residents with European ancestry) population (this QC step was performed upon merging with the WTCCC dataset as explained below). SNPs genotyped in the primary analysis cohort were excluded if any of the following criteria were met: a) minor allele frequency (MAF) <0.01, b) there was deviation from Hardy-Weinberg Equilibrium (HWE), taken as p<0.0001 or c) genotyping success rate was <95%. All QC analysis was undertaken using PLINK v1.07 [12] unless otherwise stated. The same SNP quality control criteria were also applied to the WTCCC and both of the validation cohorts.

The overlapping SNPs in the primary analysis cohort genotypes with WTCCC dataset were merged. Before merging, any A/T and G/C SNPs were removed to avoid strand flip issues. The merged dataset was then subject to SNP phasing using SHAPEIT [14] and imputation using IMPUTE2 [15,16] being undertaken using the 1000 genome phase 3 reference panel (March 2012 release). After imputation, imputed SNPs with INFO score (which indicated imputation quality) <0.5 and MAF <0.01 were removed from the subsequent genome-wide analysis.

Statistical Analysis

In the primary analysis cohort, to test for association, regression models assuming an additive genetic model were fitted in SNPtest using each SNP as a covariate in an independent model. To adjust for population substructure we included up to five principal components as covariates in this genome-wide analysis, subject to the principal components being significantly associated with the outcome univariately (p<0.05). If no principal components were significantly associated with the outcome, the first two principal components were included as covariates in genome-wide analysis. Previously in Hawcutt et al. (2015)[2], the cumulative dose in the past 6 months was identified as significantly associated with impaired peak cortisol (see supplementary data section). However, due to the inclusion of WTCCC in the control group, cumulative dose could not be adjusted for in the genome-wide analysis of binary outcomes.

Secondary analyses were also undertaken within the primary analysis cohort to test for association with peak cortisol levels and baseline cortisol levels, both as continuous phenotypes. Baseline cortisol level data were logarithmically transformed to achieve normality. Tests for association between each SNP in turn and phenotype were undertaken by fitting linear regression models in

SNPtest, again with two principle components included as covariates. A covariate to adjust for total corticosteroid dose and age was also included in the regression models for peak and baseline phenotypes respectively. These covariates were chosen based on our previous findings[2]. In all analyses, as an additive genetic model was assumed, all odds ratios reflect the comparison between heterozygotes versus wild-type homozygotes.

Selection of genes for validation

Any SNPs located within genes (including all promoter and intronic regions) identified in the primary analysis cohort, with a p value $<1x10^{-6}$ for association with any of the phenotypes were considered for validation. Commonly, the threshold of $1x10^{-5}$ is used in genome-wide analysis as a threshold of "nominal significance", but for this study we considered the lower threshold of $1x10^{-6}$ to reduce the risk of identifying false positive results whilst taking into consideration the small size of the PASS cohort.

If multiple SNPs achieved significance, then the decision on which to undertake validation was based on the presence of genotyped SNPs, the credibility of the LocusZoom plot (i.e. a reasonable LD structure of proportionality between ascending r^2 values and descending p-values), and supporting evidence of biological plausibility.

As the use of WTCCC controls can lead to false positive associations due to assuming a >350nmol/L corticosteroid level or >500nmol/L corticosteroid level response when exposed to the PASS study conditions, further analysis was conducted on SNPs (and their regions) recommended for validation using only the PASS dataset without the WTCCC controls. In this further analysis, we adjusted for significant clinical covariates (cumulative dose) and any applicable interaction effects between clinical covariates and genetic loci (if significant at the 5% level).

Genotyping of validation samples was undertaken using Taqman allelic discrimination assay C___1951874_10 (Thermo Fisher Scientific, Paisley, UK) according to the manufacturer's protocol. All individuals were genotyped in duplicate and an absolute requirement for concurrence between the 2 samples, as well as a genotyping call rate of >95% and a Hardy Weinberg p-value of >0.0001 were applied, for quality control. Statistical analyses of SNPs selected for validation were conducted in R[17] repeating the same methodology as that used for genome-wide analysis, i.e. logistic regression with binary phenotypes and linear regression with continuous phenotypes with adjustment for the same covariates as the genome-wide analysis.

Comparison with the CORNET consortium data

The association between rs591118 and morning plasma cortisol was investigated in 12 studies that participated in the discovery and replication stages of the CORtisol NETwork (CORNET) consortium genome-wide association meta-analysis[18] and in four additional studies that have recently joined the CORNET consortium. The total dataset included data from 16 studies and a total of 24,467 individuals of European ancestry: Orkney Complex Disease Study (ORCADES), Croatian Biobank (Korcula, Split, Vis), Rotterdam Study (RS), Helsinki Birth Cohort 1934-44 (HBCS), North Finnish Birth Cohort 1966 (NFBC1966), Avon Longitudinal Study of Parents and Children (ALSPAC), Prospective Investigation of the Vasculature in Uppsala Seniors (PIVUS), Prevention of Renal and Vascular End-stage Disease (PREVEND), Western Australia Pregnancy Cohort (Raine), Osteoporotic Fractures in Men – Sweden (MrOS), TwinsUK, Cooperative Health Research in the Augsburg Region (KORA), Viking Health Study – Shetlands (VHSS) and Study of Health in Pomerania (SHIP). The meta-analysis was conducted using METAL software[19].

Pathway Analysis

We used MAGENTA[20] to scrutinise the results from all phenotypes to identify any enrichment of functional and biological pathways. SNPs were annotated to genes if they fell within a 5kb boundary surrounding a gene (including all promotor and intronic regions) [21]. The pathways and ontology terms used in MAGENTA are from Biocarta, KEGG, Ingenuity, Panther, Reactome and Gene Ontology (GO) databases. We refined the databases down to the pathway and ontology terms that included the genes that had been identified through annotation of the SNPs selected for validation.

Pathways were not limited to gene set size. A nominal p-value for Gene Set Enrichment Analysis (GSEA) was calculated through permutation via random resampling of 10,000 gene sets of identical size. Both MAGENTA thresholds for gene-level association signals were utilised (75th and 95th percentile), to ensure that weaker gene-level association signals could still be investigated. The FDR threshold of 5% was chosen across all pathway analyses to indicate a significant pathway.

The Ingenuity® Pathway Analysis software (IPA®, QIAGEN Redwood City), which is powered by manually curated literature within the Ingenuity Knowledge Base, was utilised to explore the known gene networks and regulatory cascades that lead to or from our identified gene.

Results

Review of Publications looking for biological plausibility

Publications for *PDGFD* and *TRPA1* were reviewed to examine possible biological plausibility. There were no relevant publications noted for six of the eight genes. *TRPA1* has been associated with Familial Episodic Pain Syndrome[22], and *TRPA1* expression (after painful stimuli) has been linked to increased HPA axis responses in mice[23]. Pain is well recognised as a stimulus to the HPA axis, as part of its normal function. In relation to *PDGFD,* PDGF receptors are required in the development of steroid-producing cells in multiple organs, including the testis, ovary, and adrenal cortex[24]. In addition, expression of *PDGFD* has been negatively correlated with cortisol secretion in adrenocortical adenomas[25]. This was the only publication from any of the genes related to altered cortisol secretion.

Pathway Analysis

Sixteen pathways were included in a pathway analysis that contained the *PDGFD* gene. There were two pathways identified that were associated with a FDR corrected p value <0.05 (see supplementary data). These were Focal Adhesion and Regulation of Actin Cytoskeleton, both from the KEGG database. We also used IPA® to evaluate the biological interactions and pathways known to encompass *PDGFD*. Within the canonical PDGF signalling pathway involving *PDGFD*, several cytoplasmic signalling proteins are recruited, leading to the activation of a number of key signalling cascades including the mobilization of cytosolic calcium and mitogen activated protein kinase (MAPK) pathway.

Number needed to test full methodology

For children with asthma prescribed ICS, we first calculate the Number Needed to Test (NNT) in the PASS replication cohort, as the effect size can be considered unbiased. Considering the rs591118 genotype within the PASS validation cohort, using the <350nmol/L definition of adrenal suppression, the frequency of adrenal suppression in the wild-type homozygous (GG) group is 0.029 (2.9 per 100); heterozygous (AG) group is 0.097 (9.7 per 100); mutant-type homozygous group (AA) is 0.3 (30 per 100). The frequency of the heterozygous genotype is 0.41, and that of the homozygous mutant-type genotype is 0.13. Assuming that we would use non-steroid treatment in heterozygotes and mutant homozygotes, we avoid 9.7 events per 100 paediatric patients in the heterozygous group and 30 events per 100 patients in the mutant-type homozygous group. This translates to needing to stop

treating 11 ([1/9.7]*100) patients from the heterozygous group and 4 ([1/30]*100) patients from the mutant-type group to stop one event. The NNT to identify a sufficient number of children at increased risk of adrenal suppression and avoid one event of adrenal suppression would therefore be 27 (11/0.41) for heterozygous patients (95% CI 5-Inf) and 31 (4/0.13) for mutant-type homozygous patients (95% CI 16-485). Taking the lowest of these values, we need to test 27 (95% CI: 5-Inf) patients.

Collating all the data from paediatric cohorts provides a more precise estimate of NNT but may be subject to a potentially inflated effect size derived the derivation cohort. Considering the rs591118 genotype within the PASS discovery and validation cohorts combined. Using the <350nmol/L definition of adrenal suppression, the frequency of adrenal suppression in the wild-type homozygous (GG) group is 0.017 (1.7 per 100); heterozygous (AG) group is 0.023 (2.3 per 100); mutant-type homozygous group (AA) is 0.182 (18.2 per 100). The frequency of the heterozygous genotype in the two cohorts combined is 0.45, and that of the homozygous mutant-type genotype is 0.16. Assuming that we would use non-steroid treatment in heterozygotes and mutant homozygotes, we avoid 2.3 events per 100 paediatric patients in the heterozygous group and 18.2 events per 100 patients in the mutant-type homozygous group. This translates to needing to stop treating 44 ([1/2.3]*100) patients from the heterozygous group and 6 ([1/18.2]*100) patients from the mutant-type group to stop one event. The NNT to identify a sufficient number of children at increased risk of adrenal suppression and avoid one event of adrenal suppression would therefore be 98 (44/0.45) for heterozygous patients (95% CI 54-740) and 38 (6/0.16) for mutant-type homozygous patients (95% CI 24-61). Taking the lowest of these values, we need to test 38 (95% CI: 24-61) patients.

For adults with COPD: For the rs59118 genotype within the PASIC validation cohort, using the <500nmol/L definition of adrenal suppression, the frequency of adrenal suppression in the wild-type homozygous group is 0.11 (11 per 100); heterozygous group is 0.21 (21 per 100); mutant-type homozygous group is 0.41 (41 per 100). The frequency of the heterozygous genotype in the cohort is 0.44, and that of the homozygous mutant-type genotype is 0.22. Assuming that we would use non-steroid treatment in heterozygotes and mutant homozygotes, we avoid 21 events per 100 paediatric patients in the heterozygous group and 41 events per 100 patients in the mutant-type homozygous group. This translates to needing to stop treating 5 ([1/21]*100) patients from the heterozygous group and 3 ([1/41]*100) patients from the mutant-type group to stop one event. The NNT to identify a sufficient number of children at increased risk of adrenal suppression and avoid one event of adrenal suppression would therefore be 12 (5/0.44) for heterozygous patients (95% CI 7-23) and

14 (3/0.22) for mutant-type homozygous patients (95% CI 9-23). Taking the lowest of these values, we need to test 12 (95% CI: 7-23) patients.

Supplementary Table 1

Peak cortisol	PASS & EMSC patients		PASS patients		All	
**Independent Variable**	**n**	**p-value**	**n**	**p-value**	**n**	**p-value**
Age	279	0.84	152	0.70	432	0.44
Gender	279	0.008	151	0.93	434	0.09
Gender (age ≥12 years)	111	**0.002***	87	0.68	202	0.027
Inhaled/intranasal dose– no adjustment for oral/rescue	278	0.98	146	0.026	429	0.06
Inhaled/intranasal steroid cumulative dose – adjusted fororal/rescue	269	0.63	138	0.14	409	0.56
Number of courses of rescue steroids(excluding those on oral steroids; no adjustment for inhaled/intranasal)	248	0.77	124	0.16	373	0.21
Number of courses of rescue steroids (excluding those on regular oral steroids; adjustment for inhaled/intranasal)	247	0.75	121	0.32	369	0.31
Number of courses of rescue steroids (including those on regular oral steroids; no adjustment for inhaled/intranasal and oral)	270	0.35	142	0.25	416	0.11
Number of courses of rescue steroids (including those on regular oral steroids; adjustment for inhaled/intranasal and oral)	269	0.54	138	0.38	411	0.44
Total steroid cumulative dose	278	0.04	146	**0.001***	429	**<0.001***

Supplementary table 1 (adapted from Hawcutt et al [2]: *Significant following Bonferonni correction (p<0.005). Pharmacogenetics of adrenal suppression study (PASS), Early Morning Salivary Cortisol Study (EMSC). Associations with peak cortisol.

Supplementary Table 2

RS_Number	Coord	Alleles (Minor/Alternative)	MAF				INFO	R2	RegulomeDB	Peak350_PASSWTCCC			Peak350_PASS*			Peak350 _PASS$	PASSvsWTCCC
			GBR_REFERENCE	PASS_CASES	PASS_CONS	WTCCC				Beta	Standard Error	P-value	Beta	Standard Error	P-value*	P-value	P-value
**rs591118**	chr11:103966721	(A/G)	0.40	0.81	0.36	0.36	0.99	1.00	3a	-1.99	0.42	5.84E-08	-2.17	0.49	9.08E-08	1.07E-07	0.28
**rs361283**	chr11:103960620	(T/C)	0.41	0.81	0.37	0.38	0.99	0.98	7	-1.94	0.42	1.32E-07	-2.13	0.49	1.86E-07	2.53E-07	0.25
**rs603781**	chr11:103965133	(T/C)	0.41	0.81	0.37	0.38	1.00	0.98	7	-1.92	0.42	1.75E-07	-2.09	0.48	2.46E-07	3.48E-07	0.32
**rs623031**	chr11:103969890	(C/G)	0.31	0.69	0.29	0.30	0.99	0.68	6	-1.72	0.37	7.67E-07	-1.86	0.42	1.07E-06	1.31E-06	0.46
**rs618648**	chr11:103969657	(C/A)	0.31	0.69	0.29	0.30	0.99	0.68	7	-1.72	0.37	7.76E-07	-1.85	0.42	1.08E-06	1.32E-06	0.47
**rs5794293**	chr11:103969702	(CTTT/-)	0.31	0.69	0.29	0.30	0.99	0.68	NA	-1.72	0.37	7.77E-07	-1.85	0.42	1.08E-06	1.32E-06	0.47
**rs619114**	chr11:103969553	(C/T)	0.31	0.69	0.29	0.30	0.99	0.68	4	-1.72	0.37	7.81E-07	-1.85	0.42	1.08E-06	1.33E-06	0.47
**rs574494**	chr11:103969440	(A/G)	0.31	0.69	0.29	0.30	0.99	0.68	4	-1.72	0.37	7.85E-07	-1.85	0.42	1.08E-06	1.33E-06	0.48
**rs619954**	chr11:103969434	(G/A)	0.31	0.69	0.29	0.30	0.99	0.68	4	-1.71	0.37	7.85E-07	-1.85	0.42	1.09E-06	1.33E-06	0.48
**rs620426**	chr11:103969332	(C/G)	0.31	0.69	0.29	0.30	0.99	0.68	6	-1.71	0.37	7.90E-07	-1.85	0.42	1.09E-06	1.34E-06	0.48
**rs2515083**	chr11:103968747	(A/C)	0.31	0.69	0.29	0.30	1.00	0.68	6	-1.71	0.37	8.15E-07	-1.84	0.42	1.11E-06	1.37E-06	0.49
**rs517401**	chr11:103967778	(T/C)	0.31	0.69	0.29	0.30	1.00	0.68	5	-1.70	0.37	8.30E-07	-1.84	0.42	1.12E-06	1.38E-06	0.50
**rs2515080**	chr11:103967565	(T/C)	0.31	0.69	0.29	0.30	1.00	0.68	6	-1.70	0.37	8.30E-07	-1.84	0.42	1.12E-06	1.38E-06	0.50
**rs589796**	chr11:103967023	(T/C)	0.31	0.69	0.29	0.30	1.00	0.68	5	-1.70	0.37	8.30E-07	-1.84	0.42	1.12E-06	1.38E-06	0.50
**rs671851**	chr11:103968170	(A/G)	0.31	0.69	0.29	0.30	1.00	0.68	6	-1.70	0.37	8.31E-07	-1.83	0.42	1.12E-06	1.38E-06	0.50
**rs590216**	chr11:103964427	(A/C)	0.31	0.69	0.29	0.30	1.00	0.68	5	-1.66	0.36	8.35E-07	-1.83	0.42	1.12E-06	1.38E-06	0.51
**rs361284**	chr11:103961062	(G/A)	0.31	0.69	0.29	0.30	0.99	0.68	7	-1.63	0.36	8.57E-07	-1.75	0.41	1.27E-06	1.27E-06	0.39

Supplementary Table 2: Deep analysis of the 20 SNPs with a p-value of less than 1x10^-6 in the PASS+WTCCC analysis. *Adjusted for the 1st two principal components and significant clinical factor: total corticosteroid dose. $Adjusted for the 1st two principal components

Supplementary Table 3

	Database	Gene Set	#Gene	GSEA p-value	FDR Corrected (Percentile Cut-off)
Peak 350	GOTERM	Golgi membrane	350	0.12	0.23 (75)
	GOTERM	Extracellular Region	1863	0.06	0.32 (75)
	GOTERM	Growth Factor Activity	161	0.30	0.35 (75)
	GOTERM	Regulation of Peptidyl-tyrosine Phosphorylation	10	0.36	0.38 (75)
	GOTERM	Positive Regulation of Cell Division	37	0.75	0.67 (75)
	Panther Biological Process	Cell Proliferation and Differentiation	1028	0.24	0.70 (95)
	Panther Biological Process	Other Developmental Process	104	0.39	0.55 (95)
	Panther Biological Process	Ligand-mediated Signaling	421	0.90	0.90 (95)
	Panther Molecular Function	Growth Factor	118	0.53	0.53 (75)
	Panther	Angiogenesis	80	0.08	0.08 (95)
	Ingenuity	PDGF Signaling	23	0.27	0.27 (75)
	KEGG	KEGG Focal Adhesion	201	2.4×10^{-4}	6.0×10^{-4} (95)*
	KEGG	KEGG Gap Junction	90	0.07	0.09 (95)
	KEGG	KEGG Regulation of Actin Cytoskeleton	216	0.01	0.01 (95)*
	KEGG	KEGG Prostate Cancer	89	0.10	0.27 (95)
	KEGG	KEGG Melanoma	71	0.49	0.43 (95)

Supplementary Table 2: Pathways associated with the phenotypes of the PASS cohort. *Pathways with FDR corrected p-value < 0.05.

Supplementary Figure 1

Principal Component Analysis of the PASS cohort with reference datasets (Han Chinese in Beijing (CHB), Japanese in Tokyo (JPT), Yorubans in Ibadan, Nigeria (YRI) and Utah residents with Northern and Western European Ancestry (CEU)

Supplementary Figure 2

Locus Zoom of the *PDGFD* region in chromosome 11 with lead SNP rs591118 (A: Peak Cortisol 500nmol/l, B: Peak Cortisol 350nmol/l)

Supplementary Figure 3

Box Whisker plot of peak cortisol against rs591118 genotype for each cohort

Supplementary Figure 1

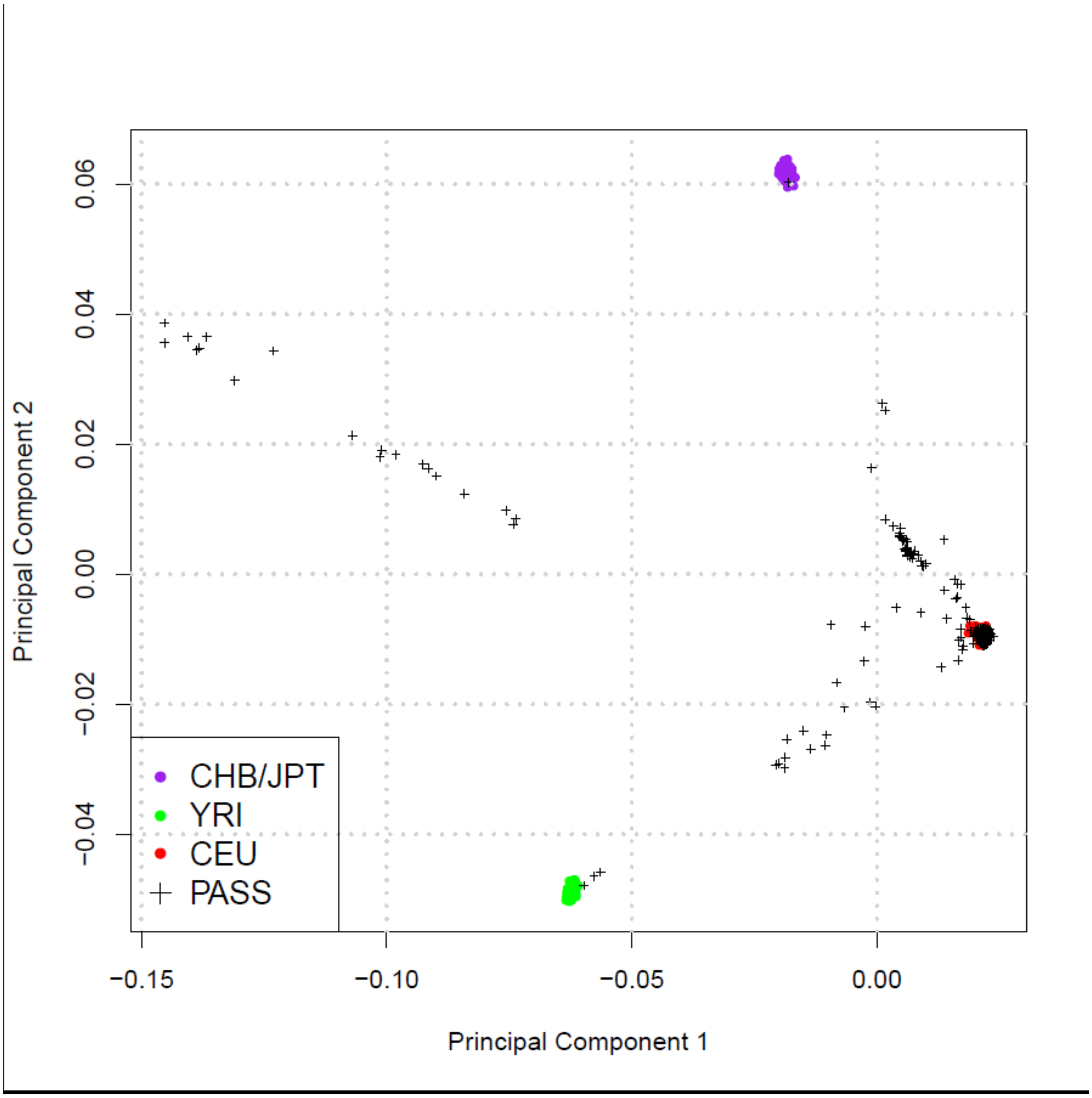

Supplementary Figure 2A

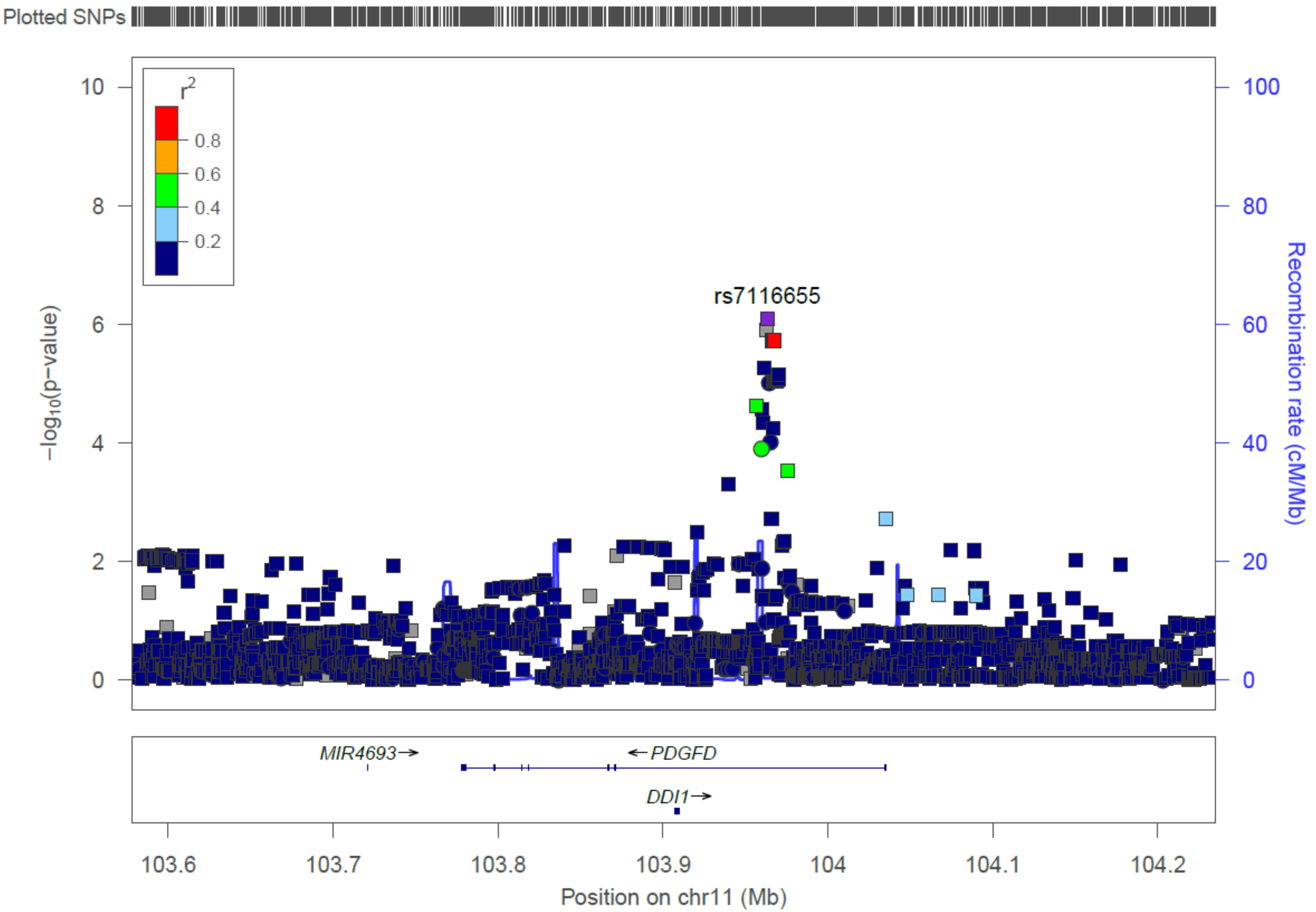

Supplementary Figure 2B

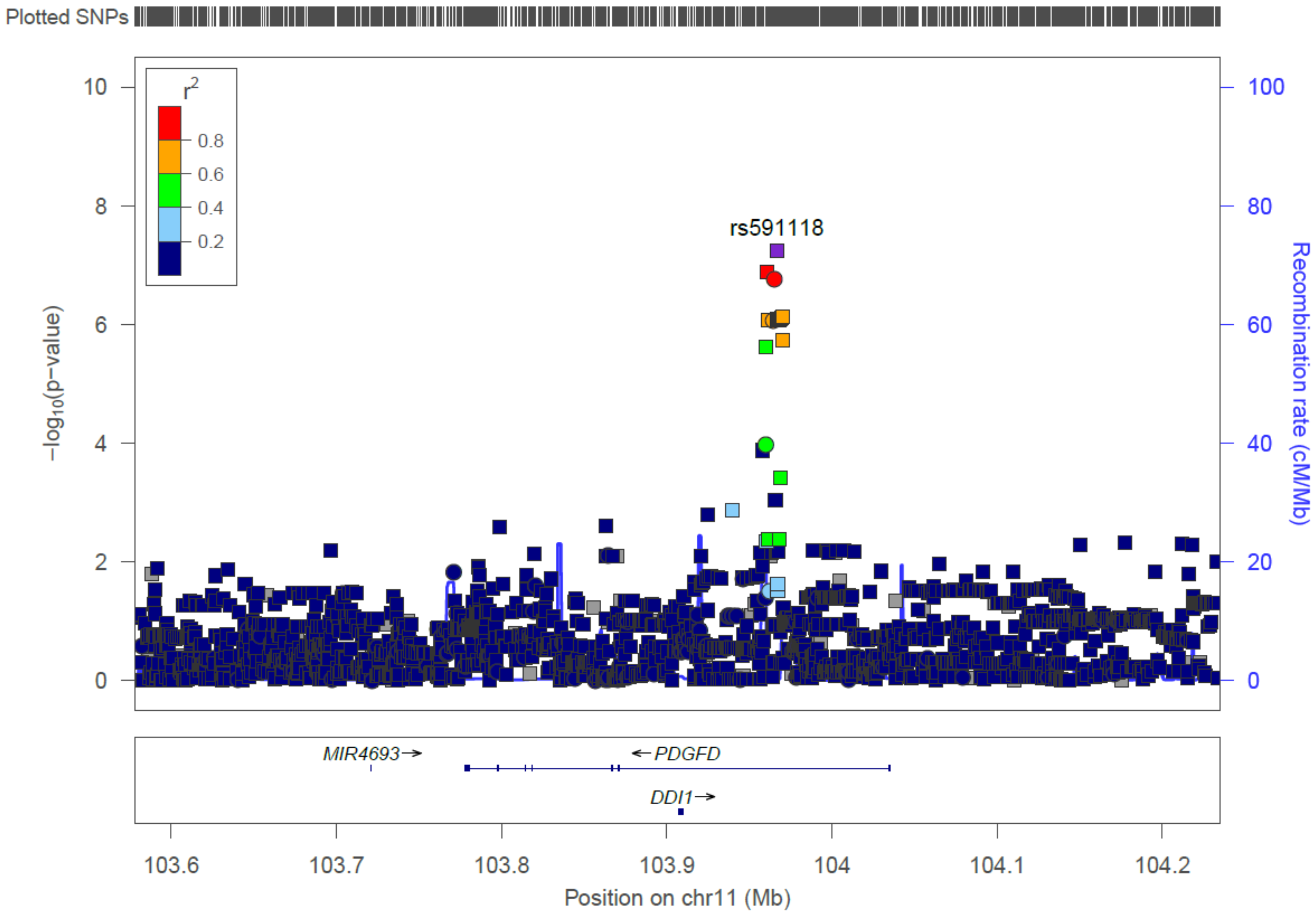

Supplementary Figure 3

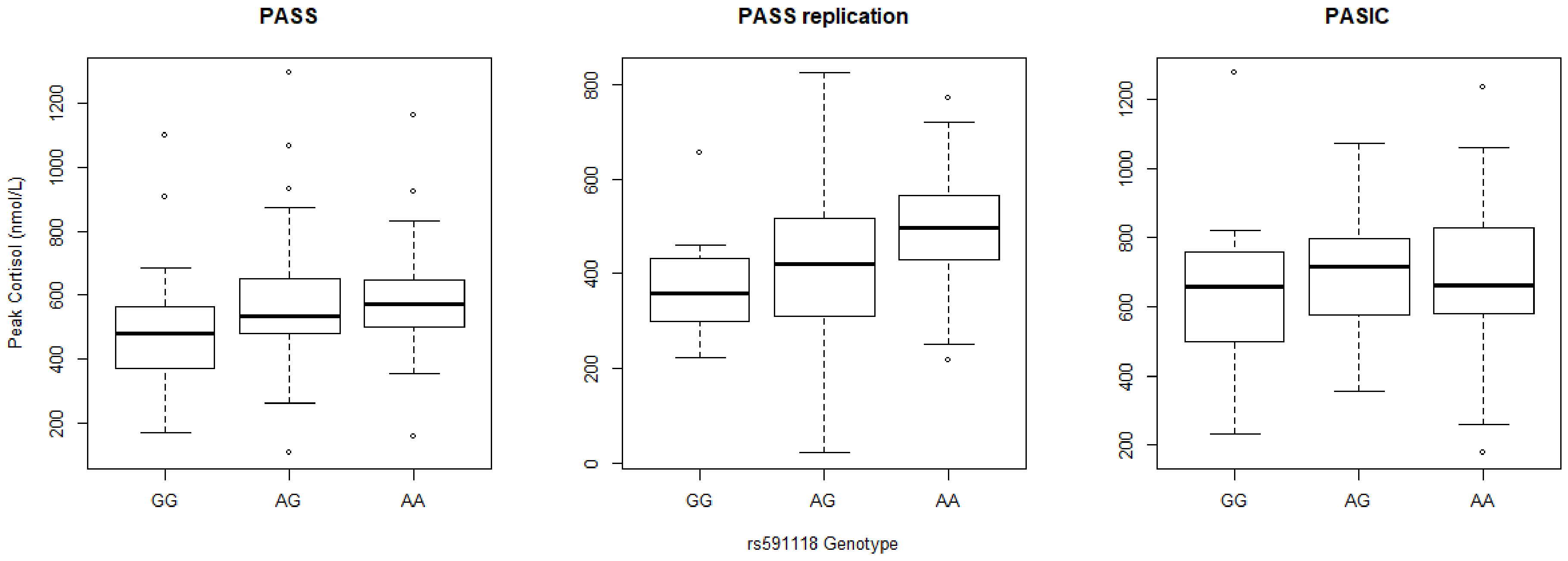

References

1. Paton J, Jardine E, McNeill E, et al. Adrenal responses to low dose synthetic ACTH (Synacthen) in children receiving high dose inhaled fluticasone. *Archives of Disease in Childhood* 2006; **91**(10): 808-13.

2. Hawcutt DB, Jorgensen AL, Wallin N, et al. Adrenal responses to a low-dose short synacthen test in children with asthma. *Clinical endocrinology* 2015; **82**(5): 648-56.

3. Zhang LJ, Axelsson I, Chung M, Lau J. Dose Response of Inhaled Corticosteroids in Children With Persistent Asthma: A Systematic Review. *Pediatrics* 2011; **127**(1): 129-38.

4. British Guideline on the Management of Asthma. In: (BTS) BTS, (SIGN) SIGN, editors.; 2007. p. 103.

5. Jennings BH, Andersson KE, Johansson SA. Assessment of systemic effects of inhaled glucocorticosteroids - comparison of the effects of inhaled budesonide and oral prednisolone on adrenal-function and markers of bone turnover. *European Journal of Clinical Pharmacology* 1991; **40**(1): 77-82.

6. Gamble J, Stevenson M, McClean E, Heaney LG. The prevalence of nonadherence in difficult asthma. *American journal of respiratory and critical care medicine* 2009; **180**(9): 817-22.

7. Network BTSSIG. British guideline on the management of asthma. *Thorax* 2014; **69**(Suppl 1): i1-i192.

8. Blair J, Lancaster G, Titman A, et al. Early morning salivary cortisol and cortisone, and adrenal responses to a simplified low-dose short Synacthen test in children with asthma. *Clinical endocrinology* 2014; **80**(3): 376-83.

9. Magnotti M, Shimshi M. Diagnosing Adrenal Insufficiency: Which Test is Best—The 1-µg or the 250-µg Cosyntropin Stimulation Test? *Endocrine Practice* 2008; **14**(2): 233-8.

10. Lekkakou L, Tzanela M, Lymberi M, Consoulas C, Tsagarakis S, Koutsilieris M. Effects of gender and age on hypothalamic–pituitary–adrenal reactivity after pharmacological challenge with low-dose 1-µg ACTH test: a prospective study in healthy adults. *Clinical endocrinology* 2013; **79**(5): 683-8.

11. Hawcutt D, Ghani A, Sutton L, et al. Pharmacogenetics of warfarin in a paediatric population: time in therapeutic range, initial and stable dosing and adverse effects. *The pharmacogenomics journal* 2014; **14** (6), 542-548

12. Purcell S, Neale B, Todd-Brown K, et al. PLINK: a tool set for whole-genome association and population-based linkage analyses. *The American Journal of Human Genetics* 2007; **81**(3): 559-75.

13. Zheng X, Levine D, Shen J, Gogarten SM, Laurie C, Weir BS. A high-performance computing toolset for relatedness and principal component analysis of SNP data. *Bioinformatics* 2012; **28**(24): 3326-8.

14. Delaneau O, Marchini J, Zagury J-F. A linear complexity phasing method for thousands of genomes. *Nature methods* 2012; **9**(2): 179-81.

15. Howie B, Marchini J, Stephens M. Genotype imputation with thousands of genomes. *G3: Genes, Genomes, Genetics* 2011; **1**(6): 457-70.

16. Howie BN, Donnelly P, Marchini J. A flexible and accurate genotype imputation method for the next generation of genome-wide association studies. *PLoS genetics* 2009; **5**(6): e1000529.

17. foundation TR. The R project for Statistical Computing. https://www.r-project.org/ (accessed 19th May 2017).

18. Bolton JL, Hayward C, Direk N, et al. Genome wide association identifies common variants at the SERPINA6/SERPINA1 locus influencing plasma cortisol and corticosteroid binding globulin. *PLoS genetics* 2014; **10**(7): e1004474.

19. Willer CJ, Li Y, Abecasis GR. METAL: fast and efficient meta-analysis of genomewide association scans. *Bioinformatics* 2010; **26**(17): 2190-1.

20. Wang K, Li M, Hakonarson H. Analysing biological pathways in genome-wide association studies. *Nature reviews Genetics* 2010; **11**(12): 843-54.

21. Aslibekyan S, Almeida M, Tintle N. Pathway analysis approaches for rare and common variants: Insights from GAW18. *Genetic epidemiology* 2014; **38**(0 1): S86-S91.

22. Kremeyer B, Lopera F, Cox JJ, et al. A Gain-of-Function Mutation in TRPA1 Causes Familial Episodic Pain Syndrome. *Neuron* 2010; **66**(5): 671-80.

23. Pierce AN, Zhang Z, Fuentes IM, Wang RP, Ryals JM, Christianson JA. Neonatal vaginal irritation results in long-term visceral and somatic hypersensitivity and increased hypothalamic-pituitary-adrenal axis output in female mice. *Pain* 2015; **156**(10): 2021-31.

24. Schmahl J, Rizzolo K, Soriano P. The PDGF signaling pathway controls multiple steroid-producing lineages. *Genes & development* 2008; **22**(23): 3255-67.

25. Wilmot Roussel H, Vezzosi D, Rizk-Rabin M, et al. Identification of gene expression profiles associated with cortisol secretion in adrenocortical adenomas. *The Journal of Clinical Endocrinology & Metabolism* 2013; **98**(6): E1109-E21.
